# Patient Perspectives on Online Health Information and Communication With Doctors: A Qualitative Study of Patients 50 Years Old and Over

**DOI:** 10.2196/jmir.3588

**Published:** 2015-01-13

**Authors:** Michelle Pannor Silver

**Affiliations:** ^1^Department of Anthropology/Health StudiesUniversity of Toronto Scarborough CampusToronto, ONCanada; ^2^Institute of Health Policy, Management and EvaluationUniversity of TorontoToronto, ONCanada

**Keywords:** health communication, Internet, online health information seeking, barriers to patient-doctor communication, adults 50 years old and over, qualitative research

## Abstract

**Background:**

As health care systems around the world shift toward models that emphasize self-care management, there is increasing pressure for patients to obtain health information online. It is critical that patients are able to identify potential problems with using the Internet to diagnose and treat a health issue and that they feel comfortable communicating with their doctor about the health information they acquire from the Internet.

**Objective:**

Our aim was to examine patient-identified (1) problems with using the Internet to identify and treat a health issue, (2) barriers to communication with a doctor about online health information seeking, and (3) facilitators of communication with a doctor about patient searches for health information on the Internet.

**Methods:**

For this qualitative exploratory study, semistructured interviews were conducted with a sample of 56 adults age 50 years old and over. General concerns regarding use of the Internet to diagnose and treat a health issue were examined separately for participants based on whether they had ever discussed health information obtained through the Internet with a doctor. Discussions about barriers to and facilitators of communication about patient searches for health information on the Internet with a doctor were analyzed using thematic analysis.

**Results:**

Six higher-level general concerns emerged: (1) limitations in own ability, (2) credibility/limitations of online information, (3) anxiety, (4) time consumption, (5) conflict, and (6) non-physical harm. The most prevalent concern raised by participants who communicated with a doctor about their online health information seeking related to the credibility or limitations in online information. Participants who had never communicated with a doctor about their online health information seeking most commonly reported concerns about non-physical harm. Four barriers to communication emerged: (1) concerns about embarrassment, (2) concerns that the doctor doesn’t want to hear about it, (3) belief that there is no need to bring it up, and (4) forgetting to bring it up. Facilitators of communication included: (1) having a family member present at doctor visits, (2) doctor-initiated inquiries, and (3) encountering an advertisement that suggested talking with a doctor.

**Conclusions:**

Overall, participants displayed awareness of potential problems related to online health information seeking. Findings from this study point to a set of barriers as well as facilitators of communication about online health information seeking between patients and doctors. This study highlights the need for enhanced patient communication skills, eHealth literacy assessments that are accompanied by targeted resources pointing individuals to high-quality credible online health information, and the need to remind patients of the importance of consulting a medical professional when they use online health resources to diagnose and treat a health issue.

## Introduction

### Background

Initiatives to promote health and independence through self-care management are being developed around the world as a means of empowering patients [1-8]. Scholars and health professionals have raised concerns about using the Internet to identify and treat health issues because patients can misinterpret or misuse information, particularly in the absence of communication with a doctor [9-12]. This can occur because patients may have inadequate health literacy [13-15], inadequate eHealth literacy [16-20], or otherwise lack the ability to sort through online health-related information [21,22]. It has also been suggested that Internet-sourced health information can be difficult for patients to decipher because it comes from a wide range of different sources [23], may feature highly technical language [24], and the quality, accuracy, and safety of some health information available on the Internet can be suspect [25-27]. Researchers have also raised concerns that online health information seeking can increase patient anxiety [28-30] and be time consuming for patients [31].

While academics and health professionals have identified problems associated with patients’ use of the Internet to identify and treat health conditions, less is known about the extent to which these concerns resonate with patients. While eHealth literacy has been defined as the ability to find, comprehend, appraise, and access health information from electronic or online sources to address health-related concerns [17], an underdeveloped component involves patient awareness of the limitations of Internet-sourced health information. Awareness of the limitations associated with information sources is an important component of ensuring that patients can critically evaluate and effectively collaborate with health professionals in the context of making decisions that relate to their health [32,33]. Approximately 35% of adults in the United States report that they have used the Internet specifically to diagnose a health condition, and half follow up with a visit to a medical professional [34]. While we would like to assume that the half who follow up with a visit are those in need of medical attention and the other half safely resolve their health issues, less is known about the relationship between patient online health information seeking and communication with medical professionals.

Prior research suggests that when patients talk with a doctor about health information seeking behaviors, these conversations can be used to avoid costly repercussions that can result from delayed treatment or hazards that can occur in response to acting on inaccurate or misunderstood health information [35,36]. It is puzzling that patients who regularly use the Internet to identify and treat their own health conditions fail to engage in conversation with a health professional about their online health information seeking. Prior research points to cost as a potential barrier to communication because some patients cannot afford medical insurance or the financial expenses associated with medical consultations [37,38] and highlights reasons doctors may be averse to discussing patient-acquired online health information [27,30,36,39,40]. However, less is known about patient-perceived barriers to these conversations particularly among individuals with free access to medical care.

In support of earlier calls for research that enhances understandings of patients’ perspectives regarding online health information seeking [30,41-44], this exploratory study examines perspectives from a sample of patients 50 years old and over about using the Internet to identify and treat a health issue and about patient-provider communication. While much has been written about the perspectives that adolescents and younger people have about Internet-sourced health-related information [45-49], less is known about the perspective of adult populations. Prevalence rates for multiple chronic conditions increase starting around 50 years old [50,51], thus this age has been associated with a greater potential need for health information [52,53]. Among individuals 50 year old and over, Internet usage rates and social media usage rates have nearly doubled in recent years [54,55]. However, health literacy levels tend to decline with age [56], and inadequate health literacy has been associated with patient non-adherence and adverse disease management [57,58]. Thus, while experts have expressed a range of concerns regarding patient use of the Internet to search for health information, further research is needed to identify concerns among patient 50 years old and over and to explore how these concerns might relate to patient-provider communication.

### Objectives

This qualitative study examined patient-identified concerns about using the Internet to diagnose and treat a health issue among a sample of adults 50 years old and over. Concerns identified by participants who regularly communicated with a doctor about their online health information seeking were compared to participants who had never discussed their online health information seeking with a doctor. This study also explored barriers to and facilitators of patient-provider communication about patient searches for health information on the Internet.

## Methods

### Design and Procedures

In order to focus on potential barriers to communication beyond cost, participants were interviewed in Toronto, Canada, where all residents have access to a publicly funded health care system. Face-to-face interviews were conducted with individuals who were recruited through brochures posted in eight randomly selected neighborhoods. A consent form along with information about the study was distributed to each eligible participant. When written consent was given, interviews were conducted in public spaces that included community centers, cafes, and libraries. The process of advertising and interviewing participants who met the selection criteria was repeated until data saturation was achieved [59].

All participants completed a questionnaire designed for this study with a set of close-ended demographic and Internet use questions. The demographic portion included questions about participants’ gender, age, marital status, country of birth, education, and income. Internet use questions asked about the number of computers available in the participants’ home, whether the participant owned a touch screen device that had Internet access, the number of hours per week the participant spent using the Internet, how they first learned to use the Internet, whether the participant had ever used the Internet to search for information relating to the treatment of a disease or illness instead of going to the doctor, whether the participant had ever used the Internet to get information about a health issue and then felt more prepared or comfortable when speaking with a doctor, whether they had ever looked up information about a disease, illness, or injury after being diagnosed by a doctor, and whether they would feel comfortable recording their own health information online.

After completing the questionnaire, all participants participated in face-to-face interviews where they were asked a set of semistructured interview questions that explored their views on using the Internet to identify and treat a health condition and their experiences regarding communication about their online health information seeking. Participants who had never communicated with a doctor about their online health information seeking were also asked about potential barriers to communication. The interview guide is available in Multimedia Appendix 1. Ethical approval for this study was obtained from the University of Toronto, Office of Research Ethics.

### Participants

Eligibility criteria for participants included (1) community dwelling, (2) 50 years old and over, (3) fluency in English, (4) primary residence in Toronto, (5) regular contact with a primary care physician, general practitioner, or family doctor, and (6) regular use of the Internet to identify and treat health issues. In total, there were 56 participants. A subset of 14 had never communicated with a doctor or health professional about their online health searches. The interviews lasted 52 minutes on average (range 40-77). The data sources generated for analyses were verbatim transcripts of the audio-recorded interviews and synthesis of debriefing notes made during the interviews with additional summary notes made after each interview.

### Analysis

Transcripts and notes were read repeatedly before coding to focus on preserving as much detail as possible. Responses were managed and analyzed in NVivo 9 (QSR International). Participant demographic and Internet usage information were stored and aggregated using Stata 10. Using inductive content analysis [60] derived from a grounded theory approach [61], concerns regarding use of the Internet to identify and treat a health condition were initially coded as a set of statements that synthesized participants’ concerns. These initial descriptions were derived through a process that involved reading the relevant portion of each transcript multiple times, in some cases referring back to the original audio file, looking for overlap between participants, and combining ideas to form a broad set of basic descriptive statements. The author and 2 research assistants independently followed these steps, met multiple times to compare and discuss the statements, and then developed the initial descriptions through consensus.

Higher-level themes where then developed into general concerns by the author and a research assistant based on analysis of the initial descriptions and reexamination of the context in which these statements were made through the original transcripts. Interrater agreement was .786 (SE .025) calculated using Cohen’s kappa. Examples of how each of the general concern categories were operationalized is available in Multimedia Appendix 2.

After the initial descriptions and general concern categories were created, this information was quantitized [62,63] by counting the frequency of concerns expressed by participants based on whether they had ever communicated with a doctor about their online health information seeking. The proportion of total responses in each general concern category and the average number of comments per individual within each general concern category were calculated separately for participants who communicated with a doctor about online health information and those who did not. This information was included to observe whether one group was in fact more verbal or able to articulate more concerns. Then substantive differences between the two groups were analyzed.

Thematic analysis [64,65] was conducted to explore potential barriers to and facilitators of communication. Emergent themes regarding barriers to communication were initially developed through analysis of responses to the question, “Can you tell me about any concerns you might have about using the Internet to diagnose and treat a health issue?” for participants who had never communicated with a doctor about their online health information seeking. Then transcripts for these participants were examined in their entirety. Responses were initially examined separately for each participant, then similarities across participants were developed into communication barrier themes. In a similar manner, facilitators of communication between patients and doctors about online health information seeking initially emerged based on analysis of comments in response to the question, “Have you ever talked with your doctor about information you found on the Internet that relates to your health?”

## Results

### Overview

Semistructured interviews were conducted with 56 participants, 30 of whom were women (Table 1). Participants ranged in age from 50-87 years (mean 69 years). Just under half of the sample was married (46%, 26/56), and 57% (32/56) were born in Canada. In total, participants were born in 26 different countries*.* Many were well educated, and almost half of the sample had a household income over CAN $60,000. The majority of participants reported that they used a touch screen device (80%, 45/56), while less than half had at least one computer at home (39%, 22/56). On average, participants spent 13 hours per week using the Internet. Participants most commonly had learned how to use the Internet from a family member; 34% (19/56) of the total sample had first learned to use the Internet in a school setting. Overall, less than half of the total sample had ever used the Internet to search for health information instead of going to the doctor and then felt more comfortable talking with their doctor, and 46% (26/56) searched after being diagnosed with a health condition by their doctor. Just under half (48%, 27/56) of the sample reported that they would feel comfortable recording their own health information online.

Of the 14 participants who did not report any communication with a doctor about their use of the Internet to search for health information, nine were men. The average age of these participants was 73 years old, half were married, born in Canada, and many had a relatively high educational attainment and income. Further, 36% (5/14) of participants who had never communicated with a doctor had a home computer, 79% (11/14) owned a touch screen device, and they reported spending on average 12.8 hours using the Internet on a weekly basis. More than half of these participants had been taught to use the Internet by a family member; 43% (6/14) had ever looked up information about a disease, illness, or injury after being diagnosed by a doctor. Just under half had ever used the Internet to search for health information instead of going to the doctor or used it after being diagnosed with a health condition by their doctor. Half said they would feel comfortable recording their own health information online.

### Concerns With Using the Internet to Diagnose and Treat a Health Issue

In total, participants raised 300 potential concerns about using the Internet to diagnose and treat a health issue. These concerns were aggregated into six different higher-level general concern categories. These general concerns as well as the initial descriptive categories and frequencies are reported in Table 2. Every participant contributed to at least two different initial descriptions and at least two different general concerns. Frequencies are distinguished based on whether participants had ever discussed their online health information seeking with a doctor.

The average number of initial descriptive statements regarding problems with using the Internet to identify and treat a health issue raised by participants who communicated with a doctor about their online health searches (5.64, range 3-12) was similar to those who had never communicated with a doctor about their online health information seeking (5.57, range 2-9). However, there were substantive differences in the types of concerns each group of participants raised. Of the responses from participants who regularly communicated with a doctor, 45% (19/42) reflected general concerns about the credibility or limitations of information available online and the next highest proportion of responses (21%, 9/42) dealt with concerns about limitations in their own ability to sort through or evaluate online health information (Table 3). Among participants who had never communicated with a doctor about their online health information seeking, the highest proportion of concerns were related to non-physical harm that could arise from searching online for health information (36%, 5/14) and the next highest proportion of responses from within this group dealt with concerns related to anxiety (29%, 4/14).

**Table 1:** Participant demographic and Internet usage summary statistics.

Characteristics			Total sample (N=56), n (%)	Participants who never spoke with doctors about their online health information seeking (N=14), n (%)
**Demographic summary statistics**				
	Women		30 (54)	5 (36)
	Age (in years)		69	73
	**Marital status**			
		Married	26 (46)	7 (50)
		Divorced/Separated	10 (18)	1 (7)
		Single	8 (14)	2 (14)
		Widowed	12 (21)	4 (29)
	Born in Canada		32 (57)	10 (71)
	**Education**			
		<High school	7 (13)	3 (21)
		Completed high school	11 (20)	4 (29)
		College/University	14 (25)	4 (29)
		Graduate school	24 (43)	3 (21)
	Income over CAN $60,000		26 (46)	7 (50)
**Internet use summary statistics**				
	Has at least one computer at home		22 (39)	5 (36)
	Owns a touch screen device that has Internet access		45 (80)	11 (79)
	Hours per week spend using the Internet, in hours		13.12	12.8
	**First learned to use the Internet**			
		Self-taught	7 (13)	2 (14)
		Learned in a school setting	19 (34)	1 (7)
		Learned at work	2 (4)	1 (7)
		A friend taught me	6 (11)	2 (14)
		A family member taught me	22 (39)	8 (57)
	Ever used the Internet to search for information relating to the treatment of a disease or illness instead of going to the doctor		24 (43)	8 (57)
	Ever used the Internet to get information about a health issue and then felt more prepared or comfortable when you spoke with your doctor		23 (41)	5 (36)
	Ever looked up information about a disease, illness, or injury after being diagnosed by a doctor		26 (46)	6 (43)
	Would feel comfortable recording your own health information online		27 (48)	7 (50)

**Table 2:** Patient concerns about using the Internet to identify and treat a health issue.

General concerns	Initial descriptions	n^a^	n^b^
Limitations in own ability	I might think I am making things better when I am actually making them worse	15	2
	I might inadvertently do something that is dangerous	14	0
	I might misdiagnose the problem in the first place	10	3
	I’m afraid I won’t be able to follow the advice properly	10	2
Credibility/Limits of online information	There is a lot of low quality information out there	18	1
	I might end up chatting with someone who is not who I think it is	6	1
	I don’t believe anything that doesn’t come from my doctor's clinic	5	0
	Don’t know who the information is coming from	22	1
	It can't see me	5	0
	It can’t save me if I’ve fallen or need to go to hospital	2	0
	You can’t use it to get a prescription	3	1
	The information can be wrong	9	0
	It shouldn’t be used to replace the doctor	12	2
	It might be ok for something simple but nothing complex	9	0
	I still need to consult my doctor	8	0
Anxiety	I can end up feeling worse after I go online to look up a health problem	3	6
	Health problems start to sound so scary on the Internet	5	3
	I start thinking I have all sort of health problems	5	2
	The information can be disorienting	2	2
	It is geared toward young people and I don’t like what I find/it makes me uncomfortable	1	3
	I get overwhelmed/confused by the information	5	2
	People say unnecessarily negative things in online chat rooms, blogs, or comments sections	11	2
Time consuming	It is a waste of time	12	2
	I don’t have enough time	6	1
	I end up spending a very long time on my question	2	1
Conflict	It could upset my doctor	9	6
	It stirs up conflict with family	3	6
Non-physical harm	I may end up buying something that I shouldn’t	5	7
	My bank information may get stolen	2	4
	I could end up making a mistake, like making the wrong purchase	0	6
	I might fall into a scam and accidently send money	1	2
	I could end up getting my identity stolen	1	4
	I could get a computer virus or something that causes my computer to stop working	1	6
Total		222	78

^a^Frequencies for participants who talked with a doctor about their online health information seeking (n=42).

^b^Frequencies for participants who had never talked with a doctor about their online health information seeking (n=14).

**Table 3:** Aggregated responses in each general concern category by participant communication status.

	Proportion of total responses in each general concern category^a^	
	Participants who talked a doctor about online health information seeking (n=42), %	Participants who did not talk with a doctor about online health information seeking (n=14), %
Limitations in own ability	22	9
Credibility/Limits of online information	45	8
Anxiety	14	26
Time consuming	9	5
Conflict	5	15
Non-physical harm	5	37

^a^The cells for each column were calculated by dividing the number of initial descriptions in each general concern category by the total number of initial descriptions for participants who communicated with a doctor about their online health information seeking and separately for participants who had never communicated with a doctor about their online health information seeking.

### Barriers to Communication With a Doctor

#### Overview

A range of barriers to communication arose from the in-depth interviews. These concerns included fear of embarrassment, a feeling that the doctor does not want to hear about it, the feeling that there is no need to bring it up, and some participants could not seem to remember to bring it up.

#### What Would My Doc Think of Me?

Participants often expressed the concern that they did not understand the health-related information they found through their Internet searches and therefore did not mention these searches to a doctor. They expressed a sense of being unsure how to explain the information they found or how it related to their own condition. Participants also made sense of their decision not to discuss their online searches based on the notion that this would result in embarrassment. One participant said:

I probably don’t talk to my doctor about things I find on the Internet because I would end up making a fool of myself. I waste so much time searching around about something. I just get something in my head and my doctor doesn’t need to know about that. Most of the stuff I’m looking up is hogwash anyway, but I pretty much always end up getting confused because I’ll start with one thing and end up fifteen kilometers away from that looking up something else. What would my doc think of me if I explained how I waste my time?

85-year-old man

Online health information seeking was described as something that could be endless. Some participants discussed the time they wasted trying to find answers that only led to more questions or confusion over what they had initially suspected to be their problem, as one man described: “It’s just pointless how much information is out there. I try to get to the bottom of something and then I realize I don’t even know where to begin” [68-year-old man].

A number of participants mentioned multiple search engines and a range of health websites, but few expressed confidence in a trusted website or reliable source of information. Confusion about information read online coupled with the fear of what a doctor might think were barriers to obtaining clarification about questions that arose for some participants as they searched for health information on the Internet. This was the case, even among participants who used the same health-related websites regularly and who had relevant questions for their doctor. For example, one participant discussed how a fear of embarrassment inhibited communication:

I am embarrassed to admit it but my favorite website is this health one that sells things…I’d really like to understand how certain foods affect my prescriptions. But if I started to talk with my doctors about this website, I think they would think I was out to lunch. Or obsessed with food. I’m not going to bring it up. I don’t get most of it anyway.

86-year-old woman

This participant had a relevant question for her doctors, namely about the relationship between her prescription drugs and what she eats. However, the concern that she could not explain the information she came across, coupled with the idea that the doctors would think less of her kept her from having this conversation with her doctor.

#### My Doctor Doesn’t Want to Hear About That

Many participants showed deference to their doctors and some even implied that telling their doctor about their online health searches would be insulting to the doctor. Several participants wondered why a doctor would want to hear about what was on the Internet and feared that bringing up their online health information seeking would be insulting to the doctor. They seemed to trust that their doctors know what to look for. One participant mentioned, “Doctors can look things up on the Internet and they don’t need patients to tell them how to do that” [70-year-old man].

Another participant indicated that doctors do not want patients to tell them how to do their job:

The Internet can’t write me a prescription or look in my ear, why would I tell my doctor what I read online? Like I know what my doctor should be doing? How would that help? My doctor doesn’t want to hear about that, he wants to take some tests and check on my prescriptions.

69-year-old man

Some participants who suggested that their doctor would not want to hear about their online searches pointed to someone who had advised against this behavior. More than one participant mentioned that a daughter had told them not to discuss online searches with the doctor. These participants often described a situation where they seemed to have a specific question or set of questions but they were discouraged from bringing the discussion to the doctor for fear that “The doctor will get annoyed and he doesn’t want to hear about that” [64-year-old man].

One woman described her sense that discussing online searches with a doctor was simply not appropriate: “Oh, I love the Internet! I use it all the time…But I don’t ask my doctor about things I find online. I have a lot of friends who do that. But I know not to do that. My daughter told me they don’t like it when you do that” [84-year-old woman].

#### I Go to the Internet Instead of the Doctor

An underlying theme that emerged for some participants was that simply taking the advice they found online negated the need to discuss it. One participant mentioned a strong desire to avoid the doctor and hospital as much as possible. This participant went on to explain how easy it is to get sick in medical offices and expressed his preference for online information instead of seeing or talking with a doctor. Another participant expressed anger and frustration with his doctor. He thought that seeing his doctor was a waste of his time and implied that it would be pointless to discuss his health-related searches with his doctor:

I don’t have a relationship between looking things up online and seeing my doctor. I go to the Internet instead of the doctor. I’m tired of wasting my time with my doctor who hasn’t helped me anyway. I’m sick of waiting and I’m getting fed up quite frankly. Does it work for you?…But I do like to look things up on the Internet. I’ve been good at avoiding the clinic this way.

62-year-old man

#### It Didn’t Come up Like That

There was clearly the sense that some participants might have brought up their online health searches only if the situation was right or they could remember to bring it up to their doctor. For many participants, the idea that a doctor would want to know about their Internet health searches was surprising. At the same time, there was no indication that some participants had any intention of trying to remember to bring it up. When asked about what she did to prepare for doctor’s appointments, one participant told me that she prepared for appointments by looking up information online:

I get ready for our doctor’s appointments by looking up what we want to talk about. Sometimes I’ve made the difference in figuring out the problem. So like, when my son was complaining that his stomach was hurting, I figured out it was a hernia and I think this would have taken a long time to figure out…I wouldn’t have told them that I used the Internet research because I didn’t think about it and it didn’t come up like that.

57-year-old woman

This participant expressed a sense of self-satisfaction with the results of her online research skills. At the same time, she seemed disinclined to credit the Internet for providing the information. For other participants, the idea of bringing up information from their online searches seemed tedious. One participant said, “Oh that would be work and it is enough for me to take my meds and get to the appointments without having to make a presentation to my doctors” [70-year-old man]. This participant suggested that going to the doctor was a time when information was to be presented to him. He also made a distinction between his work, which required effort on his part, and his medical appointments, which was a time when his doctors were supposed to do the work. Another participant suggested that it simply did not occur to her to bring up the health information she had found online: “Now I hadn’t thought of that. It doesn’t come up so I just don’t know about how that conversation would go. I do look things up but my doctors probably don’t suspect and anyway I just let them tell me how to go on about things” [80-year-old man].

### Facilitators of Communication With a Doctor

#### Overview

There were three facilitators of communication between participants and their doctor(s) that emerged: having a family member present at doctor visits, doctor-initiated inquiries, and encountering an advertisement that suggested talking with a doctor.

#### Family Presence at Doctor Visits

Participants who had experience talking with a doctor about their online health information seeking often mentioned the presence of a family member at the doctor’s appointment. Family helped them remember what to ask and helped make the context for discussing their online health information seeking more comfortable. For example one man explained that “Looking at the Internet brings up a lot of questions…When my son is there, we talk with the doctor about the questions. I think this is the way people get information these days” [71-year-old man].

Family members also helped participants keep their records organized and keep track of concerns that came up between visits. Another participant suggested that her daughter keep a list of questions related to treatment options they found online for her cancer: “I talk to him and the three of us sort through things. The information is overwhelming so you need all the help you can get” [75-year-old woman].

#### Doctor-Initiated Inquiries

It was not uncommon for participants to explain that they initially started searching for health information online because their doctors had brought up the idea and that the doctors like having these discussions. For example, “I talked with my doctor about managing my diabetes and he told me about this app…When I find something, some new information I tell him. Sometimes I’ll print it out and we discuss it…He appreciates it” [74-year-old woman].

Several participants stated that they talked with their doctors about their online health information seeking when their doctor asked them directly whether they had looked online for information about their specific health condition. Another participant said, “My doctor welcomes this and we talk about things I find online at almost every visit” [56-year-old woman]. For these participants, discussions about their online health information seeking were facilitated by having doctors that inquired about patient-acquired information and who were responsive to the information patients brought them.

#### Encountering an Advertisement That Suggested Talking With a Doctor

It was also clear that advertisements encouraged some participants to initiate conversations with their doctors about their online health information seeking. For example, one participant described how she had been referred by a friend to look at a specific advertisement for a medication to treat a health condition she had:

I talked with my doctor after that probably because the ad said to. I found it online, well my friend did, and it seemed like the right fit so I brought it in with me and we ended up talking about how to look things up. My doctor even gave me a sheet.

73-year-old woman

Several participants who had brought Web-based advertisements that contained information they deemed relevant to their own health condition described how this prompted a conversation with their doctor and resulted in the transfer of information about how to look for quality Web-based resources from their doctor.

## Discussion

### Principal Findings

While prior research has warned of inherent dangers associated with having laypeople access health information online [66,67], this study contributes to existing research by describing patient-identified concerns from a sample of adults 50 years old and over. In spite of earlier conceptual work claiming that “digital immigrants”, or people not born into the digital world, are less likely to be comfortable with and use the Internet relative to younger individuals [68], participants overall were aware of a broad range of concerns about using the Internet to diagnose and treat a health issue. Many of the concerns that participants raised were similar to those that have been articulated by scholars and health professionals. However, it should be noted that this sample was educated, of higher income, and computer literate.

Although the average number of concerns raised by participants who regularly communicated with a doctor about their online health information seeking was nearly the same as participants who had never discussed their online health information seeking with a doctor, there were qualitative distinctions in the types of concerns raised by each group. Had respondents who communicated with their doctors raised more concerns on average compared to those who had never communicated with a doctor about their online health information seeking, one could have argued that the communicating group was simply more verbal or somehow able to articulate more concerns. Instead, the highest proportion of participants who regularly communicated with a doctor about their online health information seeking raised concerns about the credibility of or limitations in health-related online information sources. In contrast, the highest proportion of participants who had never discussed their online health information seeking with a doctor mentioned non-physical harm as a concern.

This substantive distinction is disconcerting because non-physical harm is inherently a non-medical issue that could arise from any use of the Internet. This evidence highlights the need to foster awareness of the fact that not all online health resources are credible and that there are physical consequences associated with misuse of online health information. While there are many incentives to promoting patient use of online health resources, this study highlights the fact that some individuals fail to recognize distinctions in the quality and credibility of different online health information resources. Thus, it supports the need for continued research that assesses eHealth literacy [69,70] in a way that also points them to high-quality online health resources.

Thematic analysis generated a set of barriers to and facilitators of communication that point to ways of enhancing the exchange of information about online health information seeking between doctors and patients. In contrast to prior research with individuals suggesting that at around 50 years old adults are less trusting of the Internet as a source of health information [71] and more inclined to trust information provided by a doctor [72,73], evidence from this study points to some patients who “go to the Internet instead of the doctor”. For these autonomous participants, prior frustrations seemed to be the overriding reason for failing to communicate and a potential barrier to future non-emergency medical visits. For these individuals and in light of potential breakdowns in patient-provider communication [74-77], it is all the more important that efforts to enhance eHealth literacy focus on potential physical dangers associated with delayed or self-treatment.

In sharp contrast were patients who were concerned that their doctor did not want to hear about their online health information seeking. Some seemed to hold their doctor in high regard and were afraid of saying the wrong thing to their doctor. This underscores prior research on the hierarchical relationship between doctors and patients [78,79]. It also provides evidence that some patients suppress communication with their doctors in the face of confusion, even if they have a practical question for their doctor. Prior work suggests that communication between doctors and patients can be facilitated when patients ask questions [80] and by communication skill interventions that focus on enhancing patient communication skills [81,82]. Findings from this study based on examination of the facilitators of communication between patients and doctors about patient use of the Internet for health information also demonstrate that doctors could be helpful in guiding patients to credible sources of online health-related information.

Consistent with prior research, a number of participants described how having family present at medical appointments facilitated conversations about their online health information seeking [83]. However, findings from this study suggest that family may serve as both an important conduit as well as a barrier to health information. Despite their ability to independently search for health information in order to identify and treat a health issue, several participants explained that their children had told them not to mention their online health information seeking to their doctor. This supports the need to further examine the role of family support in doctor-patient communication [84-88].

Other participants seemed to forget to bring up their online health information seeking with their doctor because it did not occur to them as something that they should be doing. Evidence from these participants may lend support for theories of uncertainty management, which suggest that failure to communicate can be due to intentional forgetting about acquired health information and other avoidance behaviors [66,89,90]. However, it is not clear whether these participants were forgetting because of lack of motivation, lack of an actual health threat, or some other reason. Yet regardless of the reasons for forgetting and even if participants were told by family not to bring up their online health information seeking, it is likely that they would respond to doctor-initiated conversations about their online health information seeking based on prior evidence of the hierarchical relationship between doctors and patients [78,79].

Overall findings support the need for patient skills interventions targeted at adults 50 and over and in some cases to the need for doctors to initiate conversations with patients about their online health information seeking. While it may be more difficult to reach individuals who are less inclined to consult their doctor than the Internet, evidence from this study based on examination of facilitators of communication suggests that support such as family presence or external prompts, such as advertisements, can encourage patient communication with their doctor about their online health information seeking. Findings from this study point to the need for doctors to be aware of their ability to direct patients to high-quality, credible online health resources and for policy makers to consider the fact that most patients do not have a medical education nor do they inherently know how to use online health information in a safe and effective way. It is not sufficient to create high-quality online health resources and tell patients that they ought to manage their own health issues. Instead, efforts must be made to guide patients, particularly those over 50 years old, to high-quality, credible resources and to remind them of the importance of consulting a medical professional when using online health resources to diagnose and treat a health issue.

### Limitations and Suggestions for Future Research

Findings from this study are limited to the sample from which they were collected. The communication barriers and facilitators presented in this study are limited to being reflective of interactions with doctors, though other health professionals such as nurses play critical roles in the exchange of health information [91]. Findings from this study point to the need for continued research that incorporates awareness of limitations associated with using the Internet to diagnose and treat health issues into assessments of eHealth literacy. In addition, it highlights the need for future research that explores the relationship between family support and patient-provider communication regarding online health information seeking.

### Conclusions

Exploration of patient concerns about using the Internet to diagnose and treat health issues is important in light of efforts to enhance independence and promote patient self-care management. Findings from this study point to a broad range of concerns about online health information seeking held by a sample of adults 50 years old and over, while highlighting distinctions based on whether or not patients had ever discussed information about their online health information seeking with a doctor. This study supports the need for patient skills interventions targeted at adults 50 and over, eHealth literacy assessments that are accompanied by targeted resources that point individuals to high-quality, credible online health information, and the need to remind patients of the importance of consulting a medical professional when they use online health resources to diagnose and treat a health issue.

## Multimedia Appendix 1

Interview guide.

## Multimedia Appendix 2

Operationalization of the general concern categories.
