# Supplementary material for: Patient Perspectives on Online Health Information and Communication With Doctors: A Qualitative Study of Patients 50 Years Old and Over
Source: J Med Internet Res. 2015 Jan 13;17(1):e19. doi: 10.2196/jmir.3588 (PMC4319073; doi:10.2196/jmir.3588)
Supplement: Supplementary file 1 [file jmir_v17i1e19_app1.pdf]

Appendix 1 – Interview Guide

1. Is there a computer in your home?
2. Do you own a touch screen device that has Internet access (like a tablet or smart phone)?
3. How many hours per week do you typically spend using the Internet?
4. How did you first learn to use the Internet?
5. Have you ever used the Internet to search for information relating to the treatment of a disease or illness instead of going to the doctor?
6. Have you ever used the Internet to get information about a health issue and then felt more prepared or comfortable when you spoke with your doctor?
7. Have you ever looked up information about a disease, illness, or injury after being diagnosed by a doctor?
8. Would you feel comfortable recording your own health information online?
9. Please tell me about a time when you used the Internet to get information that helped you figure out what kind of health issue you were having and how to treat it.
10. Did you get in touch with your doctor in this situation?
11. Have you ever talked with your doctor or a health professional about information you found on the Internet that relates to your health?
12. Can you tell me about any concerns you might have about using the Internet to diagnose and treat a health issue?

For respondents who never discuss their online health information seeking with a doctor:

13. You mentioned earlier that you've never talked with a doctor about information you found on the Internet that relates to your health, can you tell me a bit about why this is the case?
