# Supplementary material for: Patient Perspectives on Online Health Information and Communication With Doctors: A Qualitative Study of Patients 50 Years Old and Over
Source: J Med Internet Res. 2015 Jan 13;17(1):e19. doi: 10.2196/jmir.3588 (PMC4319073; doi:10.2196/jmir.3588)
Supplement: Supplementary file 2 [file jmir_v17i1e19_app2.pdf]

Appendix 2 – Operationalization of the General Concern Categories

What follows are examples of how each of the general concern categories were operationalized. The first example is a response that was coded into two initial descriptive categories: “The information can be wrong” and “It is a waste of time.” These were respectively categorized in two higher level general concerns: “Credibility/Limitations of online information” and “Time consuming.”

> *I know it can be dangerous to use the Internet to find out health questions. My sister was on a med she needed, she read online it was ok to take half the dose if she was feeling better. In some chat room probably. Well it wasn’t ok. The information she got was wrong and it’s probably wrong a lot of the time… you can’t mess around like that. I think you search around online because you think it’s gonna save you time but eventually you spend way more time… [54-year-old woman]*

The following is an example of a response that was initially coded as “I’m afraid I won’t be able to follow the advice properly” and then placed in the “Limitations in own ability” general category:

> *I'm afraid I won't be able to follow the advice properly. I get confused*

when I read labels never mind this stuff… I still look things up but I don't make sense of it. [63-year-old woman]

Concerns about anxiety were operationalized when participants mentioned worry, fears, apprehension, feeling disorientated or overwhelmed. For example the following statement was initially coded as "I can end up feeling worse after I go online to look up a health problem" and put into the "Anxiety" general concern category:

… sometimes it makes me very nervous and you know it makes me anxious because it's usually got information that in my situation usually isn't that upbeat. [69-year-old woman]

The following is an example of a response that was assigned to an initial code "It could upset my doctor" and the general category "Conflict":

My concerns are about getting into a situation where I find something and my doctor gets upset. This has happened… and there is this extra layer to sort through. [68-year-old man]

An example of a concern that was initially coded as "I may end up buying something that I shouldn't" and then put into the general category "Non-physical harm" was:

Well I like to look my arthritis up on the web, I find things ... but my concern is that you can end up shopping and that is a problem for me. It can be a problem these days because the information about your condition is together with something you start thinking you need to buy. [76-year-old woman]
